# Automated alerts and intraoperative noninvasive blood pressure monitoring gaps: a retrospective before-and-after study

**DOI:** 10.1186/s12871-026-04023-3

**Published:** 2026-06-12

**Authors:** Kohei Ikeda, Tsunehisa Tsubokawa

**Affiliations:** Department of Anesthesiology, The Jikei University School of Medicine, 3- 19-18, Nishi-Shimbashi Minato-ku, Tokyo, Japan

**Keywords:** Automated alert, Blood pressure monitoring gap, Anesthesia information management system, Before-and-after study

## Abstract

**Background:**

Intraoperative noninvasive blood pressure (NIBP) monitoring is recommended at least every 5 min during anesthesia, yet prolonged gaps between documented measurements still occur in routine practice. These gaps may reflect workflow interruptions, device-related factors, or failure to recognize outdated displayed values. We evaluated whether implementation of a vendor-embedded NIBP measurement gap alert was associated with fewer prolonged intraoperative NIBP monitoring gaps.

**Methods:**

We conducted a single-center, retrospective before-and-after study using anesthesia information management system data from January 1, 2018, to December 31, 2024. A vendor-embedded on-screen alert was implemented on February 24, 2021, and was triggered when no NIBP value had been charted for ≥ 5 min. Eligible cases were anesthesiology-managed surgical cases without arterial catheter monitoring or continuous non-invasive finger-cuff monitoring. The primary outcome was the case-level occurrence of at least one ≥ 10-minute NIBP measurement gap (gap10). Secondary outcomes were the maximum NIBP measurement interval per case (gaptime) and the occurrence of a ≥ 20% systolic blood pressure (SBP) change across the maximum NIBP measurement interval. Multivariable models adjusted for prespecified case-mix covariates and calendar time.

**Results:**

The analytic cohort included 34,233 cases, comprising 15,186 pre-alert and 19,047 post-alert cases. Gap10 occurred in 1,890 cases (5.52%): 1,014 of 15,186 (6.68%) in the pre-alert period and 876 of 19,047 (4.60%) in the post-alert period. In adjusted logistic regression, the post-alert period was associated with lower odds of gap10 than the pre-alert period (odds ratio [OR], 0.673; 95% confidence interval [CI], 0.499–0.906). In an adjusted Gamma model with a log link, the post-alert period was associated with a shorter expected gaptime (expected ratio, 0.951; 95% CI, 0.923–0.980). The post-alert period was also associated with lower odds of a ≥ 20% SBP change across the maximum NIBP measurement interval (OR, 0.796; 95% CI, 0.699–0.907).

**Conclusions:**

Among anesthetic cases without arterial catheter monitoring or continuous non-invasive finger-cuff monitoring, the alert was associated with fewer ≥ 10-min gaps, shorter maximum NIBP intervals, and lower odds of a ≥ 20% SBP change across the maximum NIBP interval.

**Supplementary Information:**

The online version contains supplementary material available at 10.1186/s12871-026-04023-3.

## Background

Intraoperative blood pressure monitoring is fundamental to anesthetic care, and major anesthesiology societies recommend assessing arterial pressure at least every 5 min during anesthesia unless circumstances preclude it [1, 2]. When invasive arterial monitoring is not used, noninvasive blood pressure (NIBP) is typically measured intermittently with an oscillometric cuff. Although anesthesiologists are generally aware of the intended NIBP measurement interval and aim to maintain regular cuff cycling, intermittent NIBP monitoring remains vulnerable to workflow interruptions and system-related failure modes. Scheduled NIBP cuff cycling can be disrupted by workflow demands (e.g., positioning and procedural steps). In addition, device and interface design may not clearly flag when the displayed reading is out of date, allowing prolonged gaps to go unnoticed [3, 4].

Observational analyses of electronic anesthesia records suggest that prolonged NIBP measurement gaps—defined as the interval between successive documented NIBP measurements—are not rare: ≥10-minutes interval—commonly used to indicate a clear deviation from the 5-minute standard—occur in approximately 1.8% to 7.1% of anesthetics [3, 4]. Such gaps may delay recognition of hemodynamic deterioration, and longer NIBP measurement intervals have been associated with a greater likelihood that hypotension is detected only after an interval has elapsed [5, 6]. Intraoperative hypotension itself is associated with adverse postoperative outcomes, and meta-analytic evidence links hypotension to increased 30-day mortality in noncardiac surgery [2, 7–10]. Thus, prolonged gaps are clinically relevant not only as a potential source of delayed recognition of hemodynamic change but also as a marker of monitoring reliability in routine care.

Near real-time AIMS-based alerts have been studied as clinical decision support tools, and prior reports have shown benefits for threshold-defined blood pressure documentation gaps or described associations between longer NIBP measurement intervals and delayed detection of hypotension [3–5]. However, the incremental value of a contemporary vendor-embedded NIBP gap alert remains uncertain in routine practice, where manual verification of NIBP cycling may already occur and gaps may arise from workflow interruptions, carryover of monitor settings from the preceding case, or failure to recognize outdated displayed values. To address this gap, our evaluation incorporated adjustment for both case mix and calendar-time trends and included a carryover-related workflow covariate. Given the substantial alarm burden in perioperative care, additional alerts should be evaluated rather than assumed to be beneficial [11, 12].

To address these uncertainties, we conducted a retrospective, calendar-time–adjusted before-and-after study to evaluate whether implementation of a vendor-embedded, workflow-integrated NIBP gap alert was associated with fewer case-level ≥ 10-minute NIBP measurement gaps after adjustment for case mix and calendar-time trends. We also assessed changes in maximum NIBP interval and the occurrence of a ≥ 20% systolic blood pressure (SBP) change across the maximum NIBP interval as a physiologic correlate of delayed recognition.

## Methods

### Study population and data source

This single-center retrospective before-and-after study was approved by the Ethics Committee of The Jikei University School of Medicine for Biomedical Research (37–183[12825], approved on 2025-07-29); written informed consent was waived with an opt-out option. All surgical cases managed by the Department of Anesthesiology during the study period (January 1, 2018–December 31, 2024) were identified in the operating room information system–linked anesthesia information management system (ORSYS; Philips, Tokyo, Japan). Patient demographics, case characteristics, prespecified covariates, and intraoperative noninvasive blood pressure (NIBP) measurements (values and timestamps) were extracted using data-search software (Vi-Pros, Philips, Tokyo, Japan). The unit of analysis was the anesthetic case. We excluded (1) cases with arterial line monitoring, continuous non-invasive finger-cuff monitoring, or labor analgesia (2), minor procedures without planned surgery (e.g., central venous catheterization), and (3) cases in which the patient entered the operating room but surgery was not performed. Eligible cases were classified as pre-alert or post-alert according to implementation of the NIBP gap alert on February 24, 2021. No formal sample size calculation was performed; all eligible cases were included.

### Intervention and exposure: NIBP gap alert

A vendor-embedded NIBP measurement gap alert was implemented in the operating room information system–linked anesthesia information management system (ORSYS; Philips, Tokyo, Japan) on February 24, 2021, and deployed simultaneously across all operating rooms. The alert was a visual, notification-only pop-up displayed on the electronic anesthesia record interface for all anesthesiology-managed cases. No auditory tone or other audible alarm accompanied the pop-up notification. During the study period, monitor hardware, monitor/AIMS software versions, the monitor interface, monitor–AIMS integration, and institutional alert-management policies remained unchanged; only the visual-only NIBP gap alert was introduced. The NIBP cycling interval was selectable on the physiologic monitor (e.g., 2.5, 5, 10 min, or longer), and the default interval (2.5 min) was unchanged at implementation. NIBP measurements were initiated on the physiologic monitor and transmitted to the AIMS for charting via the system interface. The visual-only pop-up alert was triggered when no NIBP value had been charted for ≥ 5 min from the timestamp of the most recent charted NIBP measurement; the timer continued even when NIBP monitoring was temporarily turned off (e.g., cuff removal). When the alert was triggered, it provided two user options: (1) temporary pause, which suppressed the alert but allowed it to reappear after an additional ≥ 5 min without a charted NIBP value, and (2) force terminate, which disabled further alerts for the remainder of the case. No formal training accompanied rollout. Alert displays and user interactions were not logged in the AIMS; therefore, response-to-alert data were unavailable. The primary exposure was alert period (post-alert vs. pre-alert). Throughout the study period, our institution used a standardized workflow that included verification of automated NIBP cycling at pre-anesthesia and pre-incision sign-out as part of routine care.

### Outcomes

NIBP measurement intervals were computed between the first and last charted NIBP measurements during the intraoperative anesthetic period. The primary outcome was the occurrence of a ≥ 10-minute NIBP measurement gap (gap10), defined at the case level as at least one inter-measurement interval of ≥ 10 min between consecutive charted NIBP values. The maximum such interval within each case was defined as gaptime (minutes). Secondary outcomes were (1) the maximum NIBP measurement interval per case (gaptime, minutes) and (2) the occurrence of a ≥ 20% systolic blood pressure (SBP) change across the maximum NIBP measurement interval within each case, defined as an absolute relative change of SBP of ≥ 20% across that interval (|SBP_after − SBP_before| / SBP_before ≥ 0.20), where SBP_before and SBP_after were the SBP values immediately before and after the maximum NIBP measurement interval. The ≥ 20% SBP change outcome was evaluated only in cases with both SBP_before and SBP_after available around the maximum NIBP measurement interval. This outcome was intended to capture whether a substantial SBP change became apparent only when NIBP monitoring resumed.

### Derivation of the operating-room–level preceding-case covariate

To capture a potential operational carryover mechanism related to prior monitor settings, we derived an operating-room–level covariate indicating whether the immediately preceding case in the same operating room had an arterial catheter, including the last case from the prior calendar day when applicable. Because NIBP cycling interval settings on our monitors are not reset by standby and may therefore carry over across cases, whereas power-off resets the interval to the default 2.5 min, this covariate was intended to reflect a plausible workflow-related source of prolonged NIBP intervals in the subsequent case. For each case, we identified the immediately preceding case in the same operating room using the operating-room identifier and operating-room entry time. For cases at the beginning of the electronic study window, if the immediately preceding case occurred before January 1, 2018, arterial-line use in that preceding case was determined by manual review of institutional records, yielding no missing values.

### Covariates

Covariates were prespecified based on clinical relevance and potential confounding in this before-and-after study. Patient-level covariates were age and sex. Surgery-related covariates included calendar time (derived from date of surgery), surgical department (15 categories), urgency (elective vs. emergency), and intraoperative position (supine, lateral, prone, lithotomy, and upright [including sitting, semi-sitting, and beach-chair positions]). An operating-room workflow covariate indicated whether the immediately preceding case in the same operating room (including the last case from the prior calendar day) had an arterial catheter (arterial line use in the preceding case). Anesthesia-related covariates included American Society of Anesthesiologists Physical Status (ASA-PS; I, II, and ≥ III), anesthesia type (volatile general anesthesia, total intravenous anesthesia [TIVA], regional anesthesia, and monitored anesthesia care [MAC]), anesthesia duration, and primary anesthesia provider category. Provider categories were attending anesthesiologist alone, intern with attending supervision, and resident with attending supervision; in Japan, interns correspond to postgraduate years (PGY) 1–2, residents to PGY3–5, and attendings to PGY6 or higher. “Attending alone” indicated no trainee involvement.

### Missing data and data handling

**Fig. 1:**
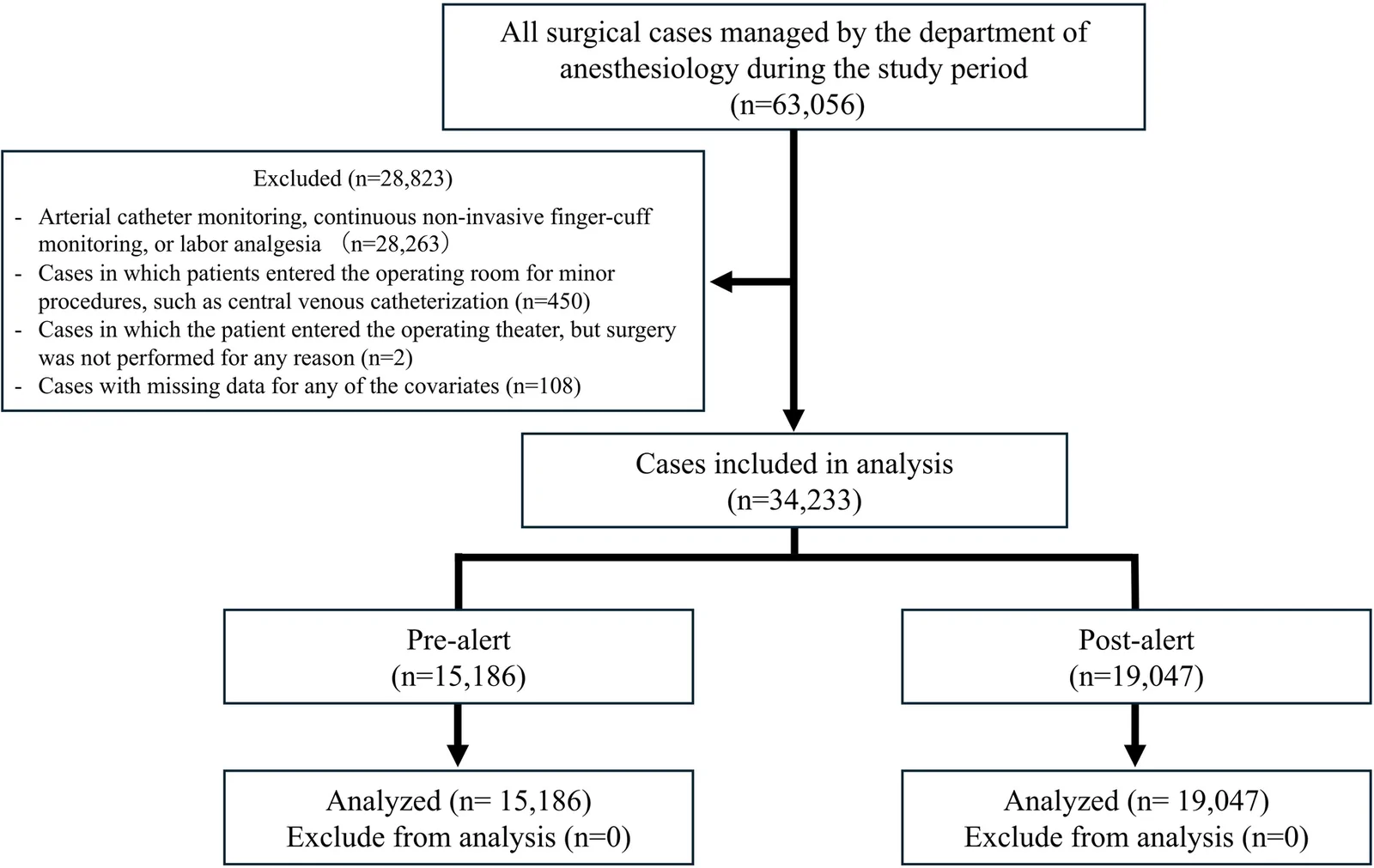
Flow diagram of case selection and exclusion criteria. Of 63,056 surgical cases managed by the Department of Anesthesiology identified in the anesthesia information management system during the study period (January 1, 2018–December 31, 2024), 28,823 were excluded: cases with arterial line monitoring, continuous non-invasive finger-cuff monitoring, or labor analgesia (*n* = 28,263), minor procedures without planned surgery (e.g., central venous catheterization; *n* = 450), cases in which the patient entered the operating room but surgery was not performed (*n* = 2), and cases with missing data for any prespecified covariates (*n* = 108). The final analytic cohort comprised 34,233 cases and was categorized into the pre-alert (*n* = 15,186) and post-alert (*n* = 19,047) periods according to implementation of the NIBP gap alert on February 24, 2021; all included cases were analyzed. NIBP, noninvasive blood pressure

Missingness was assessed for prespecified covariates used in the multivariable models. We performed a complete-case analysis, excluding cases with missing data in any covariate included in the multivariable models. No imputation was performed, and exclusions due to missing covariate data are summarized in Fig. 1. For the secondary outcome of a ≥ 20% SBP change across the maximum NIBP measurement interval, the analysis was restricted to cases with both SBP_before and SBP_after available around the maximum interval.

### Statistical analysis

All analyses were conducted at the case level in this calendar-time–adjusted before-and-after study comparing the pre-alert and post-alert periods. Baseline characteristics were summarized by period as mean (standard deviation) for continuous variables and counts (percentages) for categorical variables; between-period differences were interpreted descriptively with emphasis on standardized mean differences (SMDs). For the primary outcome, we fitted a multivariable logistic regression model for the occurrence of a ≥ 10-minute NIBP measurement gap (gap10), with alert period (post-alert vs. pre-alert) as the exposure; results are reported as odds ratios (ORs) with 95% confidence intervals (CIs). For secondary outcomes, we used models appropriate to each outcome: a Gamma generalized linear model with a log link for the maximum case-level NIBP measurement interval (gaptime), reporting exp(β) as ratios of expected gaptime, and a multivariable logistic regression model for the occurrence of a ≥ 20% SBP change across the maximum NIBP measurement interval. The latter analysis was restricted to cases with evaluable paired SBP values immediately before and after the maximum NIBP measurement interval. To assess distributional shifts in measurement intervals, we additionally fitted a multinomial logistic regression model for gaptime strata and derived adjusted predicted probabilities for each period by marginal standardization; differences are presented as post-alert minus pre-alert (percentage points). Case-level gaptime was categorized into seven mutually exclusive strata: <6, 6 to < 10, 10 to < 15, 15 to < 20, 20 to < 25, 25 to < 30, and ≥ 30 min. All multivariable models adjusted for the same prespecified covariates. Because this before-and-after design may be influenced by secular changes and period-related shifts in case mix, we adjusted for all prespecified clinically relevant covariates rather than using data-driven variable selection procedures. Calendar time was operationalized as elapsed days from study start based on date of surgery and modeled using a natural cubic spline with 3 degrees of freedom. As sensitivity analyses, we evaluated threshold-specific binary outcomes for NIBP gaps of ≥ 7, ≥10 (primary outcome), ≥ 15, and ≥ 20 min using multivariable logistic regression models with the same covariate set. We summarized the distribution of gaptime overall and by period using mean ± SD, median (IQR), upper-tail percentiles (90th, 95th, and 99th percentiles), and range (minimum–maximum). Model discrimination for binary outcomes was summarized using the area under the receiver operating characteristic curve (AUC) based on fitted probabilities. For the Gamma model, adequacy was evaluated using residual and influence diagnostics. Multicollinearity was assessed using generalized variance inflation factors (GVIFs); all adjusted GVIF values were < 4, indicating no evidence of problematic collinearity.

All analyses were performed using R version 4.5.0 (R Foundation for Statistical Computing, Vienna, Austria). Multivariable generalized linear models were fitted using the glm function (binomial family with a logit link for logistic regression; Gamma family with a log link), and calendar time was modeled using natural cubic splines (splines package, df = 3). Two-sided p values are reported for completeness; interpretation focuses on effect size and 95% CIs.

## Results

### Study cohort and baseline characteristics

A total of 63,056 anesthesiology-managed surgical cases during the study period were assessed for eligibility. Of these, 28,823 were excluded: arterial line monitoring, continuous non-invasive finger-cuff monitoring, or labor analgesia (*n* = 28,263), minor procedures such as central venous catheterization (*n* = 450), cases in which patients entered the operating room but surgery was not performed (*n* = 2), and missing covariate data (*n* = 108). The final analytic cohort included 34,233 cases (15,186 pre-alert; 19,047 post-alert), with no additional exclusions after period assignment. Baseline characteristics are summarized in Table 1 and were generally comparable between periods. The post-alert cohort was slightly older and had a somewhat higher distribution of ASA-PS categories, with modest shifts in provider composition and anesthesia type (fewer volatile and more TIVA cases), whereas other case-mix characteristics were broadly similar.

**Table 1:** Baseline characteristics of surgical cases in the pre-alert and post-alert periods

Variables	Pre-alert (*n* = 15186)	Post-alert (*n* = 19047)	SMD
Age, years (SD)	45 (23)	48 (22)	0.144
Sex, n (%)			0.019
Male	7686 (50.6)	9462 (49.7)	
Female	7500 (49.4)	9585 (50.3)	
Department of surgery, n (%)			0.107
Otorhinolaryngology	4410 (29.0)	5691 (30.0)	
Orthopedic	2480 (16.3)	3100 (16.3)	
Plastic	1535 (10.1)	1732 (9.1)	
Obstetrics and Gynecology	1487 (9.8)	1782 (9.4)	
Gastrointestinal	959 (6.3)	1113 (5.8)	
Pediatric	939 (6.2)	979 (5.1)	
Urology	737 (4.9)	984 (5.2)	
Breast and Endocrine	709 (4.7)	1005 (5.3)	
Hepato-Biliary-Pancreatic	625 (4.1)	804 (4.2)	
Ophthalmology	249 (1.6)	317 (1.7)	
Dental and Oral	236 (1.6)	398 (2.1)	
Neurosurgery	234 (1.5)	255 (1.3)	
Dermatology	184 (1.2)	159 (0.8)	
Psychiatry	162 (1.1)	471 (2.5)	
Others	240 (1.6)	257 (1.3)	
ASA-PS, n (%)			0.278
I	6779 (44.6)	5961 (31.3)	
II	7663 (50.5)	11,961 (62.8)	
≥ III	744 (4.9)	1125 (5.9)	
Primary anesthesia provider, n (%)			0.150
Attending anesthesiologist alone	3555 (23.4)	5715 (30.0)	
Resident + attending anesthesiologist	5005 (33.0)	5761 (30.2)	
Intern + attending anesthesiologist	6626 (43.6)	7571 (39.8)	
Type of anesthesia, n (%)			0.471
General Volatile	13,234 (87.2)	13,318 (69.9)	
General TIVA	1506 (9.9)	5306 (27.9)	
Regional	416 (2.7)	385 (2.0)	
MAC	30 (0.2)	38 (0.2)	
Patient position			0.063
Supine	12,543 (82.6)	16,073 (84.4)	
Lithotomy	1286 (8.5)	1612 (8.5)	
Lateral	633 (4.2)	571 (3.0)	
Prone	557 (3.7)	607 (3.2)	
Upright	167 (1.1)	184 (1.0)	
Surgical urgency			0.026
Elective, n (%)	14,299 (94.2)	18,046 (94.7)	
Emergency, n (%)	887 (5.8)	1001 (5.3)	
Duration of anesthesia, min (SD)	153 (71)	155 (73)	0.024
Arterial line use in the preceding case			0.003
Yes	3310 (21.8)	4175 (21.9)	
No	11,876 (78.2)	14,872 (78.1)	

Continuous variables are presented as mean (standard deviation, SD), and categorical variables as counts and percentages

*SMD* standardized mean difference, *ASA-PS* American Society of Anesthesiologists Physical Status, *TIVA* total intravenous anesthesia, *MAC* monitored anesthesia care

### Unadjusted incidence of gap10 and gaptime distribution

Table 2 summarizes the unadjusted incidence of gap10 (≥ 10-minute NIBP measurement gap). Overall, gap10 occurred in 1,890 of 34,233 cases (5.52%): 1,014 of 15,186 cases (6.68%) in the pre-alert period and 876 of 19,047 cases (4.60%) in the post-alert period. Descriptive distribution metrics for maximum NIBP interval (gaptime) are provided in Supplementary Table 1. The post-alert period had a lower mean gaptime (5.44 vs. 5.96 min) and lower upper-tail percentiles (P90, 7 vs. 8 min; P95, 9 vs. 10 min; P99, 15 vs. 19 min), while the median (IQR) was similar (5.00 [5.00–6.00] in both periods).

**Table 2:** Incidence of gap10 before and after alert implementation

Period	Total cases, *n*	Gap10 events, *n*	Incidence, %
Overall	34,233	1890	5.52
Pre-alert	15,186	1014	6.68
Post-alert	19,047	876	4.60

Gap10 was defined as a ≥ 10-min noninvasive blood pressure measurement gap. Values are presented as the number of gap10 events and the corresponding incidence (%) among total cases in each period

### Primary outcome: adjusted association between alert period and gap10

In the multivariable logistic regression model (Table 3), the post-alert period was associated with lower odds of gap10 than the pre-alert period (adjusted OR, 0.673; 95% CI, 0.499–0.906). Non-supine positions—particularly prone and upright—were associated with higher odds of gap10 (prone: OR, 7.081; 95% CI, 6.009–8.343; upright: OR, 4.817; 95% CI, 3.642–6.372). Longer anesthesia duration (per minute) (OR, 1.004; 95% CI, 1.003–1.004) and arterial line use in the preceding case (OR, 1.404; 95% CI, 1.258–1.567) were also associated with higher odds. Discrimination was moderate (apparent AUC, 0.712). A calibration plot is shown in Supplementary Fig. 1.

**Table 3:** Multivariable logistic regression model for ≥ 10-min NIBP measurement gaps (gap10)

Variable & Subgroup	Reference	Odds ratio	Odds ratio 95% CI (lower, upper)	*P* value
NIBP alert				
Post-alert	Pre-alert	0.673	0.499, 0.906	0.009
Age	Per 1-year increase	0.997	0.994, 1.000	0.042
Sex				
Male	Female	0.890	0.799, 0.991	0.034
Department of surgery				
Orthopedic		1.604	1.357, 1.898	< 0.001
Plastic		1.785	1.483, 2.149	< 0.001
Obstetrics and Gynecology		1.028	0.756, 1.346	0.838
Gastrointestinal		1.038	0.812, 1.327	0.765
Pediatric		1.731	1.337, 2.240	< 0.001
Urology		1.347	1.010, 1.796	0.043
Breast and Endocrine	Otorhinolaryngology	1.797	1.422, 2.271	< 0.001
Hepato-Biliary-Pancreatic		0.884	0.631, 1.239	0.476
Ophthalmology		1.612	1.039, 2.502	0.033
Dental and Oral		0.774	0.465, 1.288	0.324
Neurosurgery		1.768	1.298, 2.409	< 0.001
Dermatology		1.947	1.377, 2.752	< 0.001
Psychiatry		0.501	0.220, 1.145	0.101
Others		2.236	1.652, 3.190	< 0.001
ASA-PS				
II	ASA-PS I	1.188	1.055, 1.336	0.004
≥ III		1.623	1.314, 2.005	< 0.001
Primary anesthesia provider				
Resident + attending	Attending	1.255	1.104, 1.427	< 0.001
Intern + attending		1.319	1.156, 1.506	< 0.001
Type of anesthesia				
TIVA		1.049	0.914, 1.205	0.496
Regional	Volatile	1.444	1.106, 1.885	0.007
MAC		1.697	0.705, 4.083	0.238
Patient position				
Lithotomy	Supine	1.047	0.806, 1.361	0.728
Lateral		1.571	1.265, 1.950	< 0.001
Prone		7.081	6.009, 8.343	< 0.001
Upright		4.817	3.642, 6.372	< 0.001
Surgical urgency				
Emergency	Elective	0.737	0.582, 0.933	0.011
Duration of anesthesia	Per 1-min increase	1.004	1.003, 1.004	< 0.001
Arterial line use in the preceding case				
Yes	No	1.404	1.258, 1.567	< 0.001

A multivariable logistic regression model was used to evaluate factors associated with the occurrence of at least one ≥ 10-min inter-measurement interval in documented intraoperative NIBP measurements (gap10) at the case level. The primary exposure was the alert period (post-alert vs. pre-alert). Covariates included age, sex, surgical department, ASA-PS, primary anesthesia provider, type of anesthesia, surgical urgency, duration of anesthesia, intraoperative patient position, arterial line use in the preceding case, and calendar time modeled as elapsed days using a natural cubic spline with 3 degrees of freedom. Results are presented as odds ratios (OR) with 95% confidence intervals (CI)

*NIBP* noninvasive blood pressure, *OR* odds ratio, *ASA-PS* American Society of Anesthesiologists Physical Status, *TIVA* total intravenous anesthesia, *MAC* monitored anesthesia care

### Secondary outcome 1: maximum interval (gaptime) among all cases

In the multivariable Gamma generalized linear model with a log link (Supplementary Table 2), the post-alert period was associated with a shorter expected maximum NIBP interval (gaptime) than the pre-alert period (exp(β), 0.951; 95% CI, 0.923–0.980). Residual and influence diagnostics suggested no major violations of model assumptions and no evidence that individual observations disproportionately influenced the results.

### Secondary outcome 2: ≥20% SBP change across the maximum NIBP gap

In the multivariable logistic regression model (Supplementary Table 3), the post-alert period was associated with lower odds of a ≥ 20% systolic blood pressure (SBP) change across the maximum NIBP interval within a case (adjusted OR, 0.796; 95% CI, 0.699–0.907). Discrimination was modest (apparent AUC, 0.678).

### Distributional shift in interval strata (multinomial model)

Adjusted predicted probabilities from the multinomial logistic model showed a shift toward shorter NIBP measurement intervals in the post-alert period (Table 4). The adjusted probability of intervals < 6 min increased from 62.99% to 65.76% (+ 2.77% points), whereas the 6 to < 10-minute stratum changed minimally (30.32% to 29.69%; −0.62% points). All strata at ≥ 10 min were lower in the post-alert period (10 to < 15: −1.39; 15 to < 20: −0.34; 20 to < 25: −0.30; 25 to < 30: −0.02; and ≥ 30 min: −0.10% points), yielding a decrease in the combined adjusted probability of ≥ 10-minute intervals from 6.70% to 4.55% (− 2.15% points).

**Table 4:** Adjusted predicted probabilities of gaptime strata in the pre-alert and post-alert periods

Gaptime stratum (min)	Adjusted predicted probability Pre-alert (%)	Adjusted predicted probability Post-alert (%)	Difference (Post − Pre) percentage points
< 6	62.99	65.76	2.77
6 to < 10	30.32	29.69	-0.62
10 to < 15	4.68	3.29	-1.39
15 to < 20	0.95	0.61	-0.34
20 to < 25	0.53	0.23	-0.30
25 to < 30	0.22	0.20	-0.02
≥ 30	0.31	0.22	-0.10
≥ 10 (combined)	6.70	4.55	-2.15

Adjusted probabilities were estimated by marginal standardization from a multinomial logistic regression model including the alert period and covariates (age, sex, surgical department, ASA-PS, primary anesthesia provider, type of anesthesia, intraoperative patient position, surgical urgency, duration of anesthesia, arterial line use in the preceding case, and calendar time modeled as elapsed days using a natural spline with 3 degrees of freedom). Differences are Post-alert minus Pre-alert (percentage points)

*ASA-PS* American Society of Anesthesiologists Physical Status

### Threshold sensitivity analysis

In sensitivity analyses using multivariable-adjusted logistic regression models, the post-alert period was associated with lower odds of NIBP gaps at the ≥ 10-min threshold (events, *n* = 1,890; adjusted OR, 0.673; 95% CI, 0.499–0.906). Point estimates at the other thresholds were also below 1.0 (≥ 7 min: events, *n* = 5,997; adjusted OR, 0.905 [0.760–1.080]; ≥15 min: events, *n* = 524; adjusted OR, 0.613 [0.345–1.088]; ≥20 min: events, *n* = 232; adjusted OR, 0.581 [0.237–1.427]). Supplementary Fig. 2 displays the threshold-specific point estimates and confidence intervals. Overall, the association was most precisely estimated at the ≥ 10-minute threshold, whereas estimates at higher thresholds were less precise.

## Discussion

In this single-center, calendar-time–adjusted before–after study, implementation of a vendor-embedded NIBP gap alert was associated with lower odds of prolonged (≥ 10-minute) monitoring gaps (adjusted OR, 0.673; 95% CI, 0.499–0.906), a shorter expected maximum case-level NIBP interval (expected ratio, 0.951; 95% CI, 0.923–0.980), and lower odds of a ≥ 20% SBP change across the maximum NIBP interval within a case (adjusted OR, 0.796; 95% CI, 0.699–0.907). Taken together, these findings suggest that implementation of the visual-only alert was associated with improved monitoring reliability in routine anesthetic care, although prolonged NIBP monitoring gaps were not eliminated; the potential role of an auditory or multimodal component in addressing missed or delayed responses warrants future evaluation, with careful consideration of alarm burden and alert fatigue. In threshold-based sensitivity analyses, point estimates were also below 1.0 across alternative definitions of prolonged gaps, although estimates at higher thresholds were less precise because of fewer events.

From a practical implementation perspective, the observed association appears operationally meaningful despite the modest absolute effect size. The adjusted probability of a ≥ 10-minute gap decreased by 2.15% points, corresponding to approximately 20 fewer clear deviations from the intended 5-minute monitoring cycle per 1,000 eligible anesthetics. For institutions that use similar monitor–AIMS systems and have problems with NIBP monitoring gaps, a simple workflow-integrated visual alert may be a reasonable option. However, auditory or multimodal escalation should be evaluated cautiously because it may increase alarm fatigue or habituation, and implementation should ideally be guided by local alert frequency, user-response data, and context-aware suppression logic.

Building on these findings, our results extend prior work on AIMS-based clinical decision support. Near real-time notifications have been reported to improve perioperative process reliability, particularly documentation and protocol adherence [13, 14]. Prior studies have also described pre–post changes in threshold-defined BP documentation gaps [3, 4] and associations between longer NIBP measurement intervals and delayed detection of hypotension [5]. Our study adds to this literature in several ways. First, we evaluated the alert in a real-world, contemporary vendor-embedded implementation while adjusting for both case mix and calendar-time trends. Second, we examined not only a threshold-defined binary outcome, but also redistribution of maximum NIBP intervals across clinically relevant strata. Third, we incorporated an operating-room–level covariate reflecting arterial line use in the preceding case to capture a plausible carryover-related workflow mechanism. Fourth, we evaluated a ≥ 20% SBP change across the maximum NIBP interval as a physiologic correlate of delayed recognition when monitoring resumed. Together, these features position the present study as an implementation-focused evaluation of monitoring reliability in routine anesthetic practice rather than a simple replication of earlier alert studies.

A plausible interpretation is that the alert helped as a workflow-integrated reminder when regular NIBP cycling had been interrupted or when outdated displayed values were not immediately recognized, thereby prompting clinicians to resume cuff cycling or restore chart–device synchronization. At the same time, some prolonged gaps likely persisted because intermittent NIBP monitoring in routine practice is vulnerable to heterogeneous failure modes, including workflow interruptions during positioning or procedural steps, device or interface-related failures, and situations in which pop-up alerts are less actionable or may be intentionally suppressed [15, 16]. This interpretation is also consistent with literature showing that many perioperative alarms are nonactionable and that greater exposure to nonactionable alarms may be associated with slower responses, underscoring the importance of alert burden in real-world settings [12, 17, 18]. Context-aware delays that suppress transient, clinically expected interruptions may therefore represent one way to reduce alarm burden while preserving safety value [19]. These findings are also consistent with recent perioperative evidence from a pragmatic trial in a post-anesthesia care unit, in which customization of bedside-monitor alarm settings reduced inconsistent alarm notifications, suggesting that workflow-oriented alarm optimization may help clinicians respond more reliably to clinically relevant alarms [20]. Importantly, our aim was not to attribute each NIBP gap to a single proximate cause, but to evaluate the real-world reliability of intermittent NIBP monitoring at the case level and the association of a workflow-integrated alert with that reliability. Although calendar-time adjustment was performed, residual confounding related to unmeasured institutional or workflow changes over the study period cannot be excluded. In addition, because alert displays and user interactions were not logged, and because we could not distinguish cessation of cuff cycling from documentation or interface failures, we were unable to directly relate alert exposure or clinician responses to the observed changes [18, 21–23].

In our analysis, cases were more likely to experience a prolonged (≥ 10-minute) NIBP monitoring gap when the preceding case in the same operating room had used an arterial line (adjusted OR, 1.404; 95% CI, 1.258–1.567). One possible explanation for this association is an operational carryover effect: during arterial-line cases, clinicians may lengthen or pause NIBP cycling while relying on continuous arterial pressure, and those settings can persist into the next case if not reset. At our institution, monitors retain NIBP interval settings after standby, whereas power-off resets the interval to the default 2.5-minute setting; therefore, an extended interval from the prior case may carry over unless manually corrected after standby. Similar persistence of monitor settings and failure to re-engage automated cycling has been reported as a technical cause of prolonged NIBP intervals and BP documentation gaps [4, 5]. Few studies have explicitly evaluated inter-case carryover of monitor settings (e.g., following an arterial-line case) as a contributor to NIBP gaps in multivariable models, highlighting this as an under-studied mechanism. This observation underscores the potential value of improved equipment handoffs or system safeguards—such as automated reset of NIBP cycling between cases—to support intended 5-minute monitoring and enhance intraoperative safety. Notably, throughout the study period, our institution maintained a standardized pre-incision time-out process that included joint verification by the anesthesia team, surgeons, and operating-room nurses that NIBP measurement was active and that the cycling interval was set appropriately (typically 2.5–5 min).

Beyond the specific alert evaluated here, our study illustrates a scalable approach to assessing monitoring reliability using routinely collected AIMS data. Prior AIMS-based alerts have improved documentation and process compliance, supporting the broader potential of workflow-integrated notifications to enhance perioperative reliability [13, 14, 22, 24]. Similar outcome definitions and adjustment strategies may be extendable across institutions with relatively limited additional effort through existing benchmarking infrastructures [25, 26]. Standardized reliability metrics may reduce reliance on manual audits, although reuse of physiologic data requires careful attention to data provenance and filtering because artifacts can bias results [27–29]. A key next step is systematic logging of alert displays and user responses, which would help relate alert burden and clinician behavior to measured safety outcomes and support stronger quasi-experimental evaluations [30, 31].

Several limitations should be considered. First, internal validity is constrained by the before–after design; although we adjusted for calendar time, unmeasured time-varying co-interventions or structural changes may still have contributed to the observed differences [30]. Second, outcomes were derived from recorded NIBP timestamps rather than system logs of alert displays and user responses, and AIMS-derived physiologic data may be affected by artifacts if filtering is insufficient [29]. Because alert displays and user interactions were not logged, we could not determine how often the alert was shown, acknowledged, suppressed, or ignored, nor could we directly relate clinician responses to the observed associations. Third, external validity is limited by the single-center setting and local workflow conventions and monitor–AIMS integration. Fourth, we evaluated monitoring process measures and a physiologic correlate rather than downstream clinical outcomes. These limitations notwithstanding, the findings suggest several practical next steps, including systematic logging of alert displays and user responses, development of context-aware alert logic to limit unnecessary alarm burden, and workflow or engineering safeguards—such as improved equipment handoffs or automated reset of NIBP cycling between cases—to better support intended 5-minute monitoring in routine care [19, 30, 31].

## Conclusions

In a large cohort of anesthetic cases monitored noninvasively without an arterial catheter or continuous non-invasive finger-cuff monitoring, implementation of a vendor-embedded NIBP gap alert was associated with fewer case-level ≥ 10-minute measurement gaps, a shift toward shorter maximum NIBP intervals, and lower odds of a ≥ 20% systolic blood pressure change across the maximum NIBP interval within a case. Although prolonged gaps and substantial SBP changes still occurred, these findings suggest that implementation of the alert was associated with improved monitoring reliability in routine anesthetic care. Future efforts should focus on context-aware alert logic, systematic logging of alert displays and user responses, and workflow or engineering safeguards to further reduce prolonged monitoring gaps.

## Supplementary Information

Supplementary Material 1.

Supplementary Material 2.

Supplementary Material 3.

Supplementary Material 4: Supplementary Figure 1. Calibration of the multivariable logistic regression model predicting occurrence of gap10. Predicted probabilities from the fitted model are plotted against the observed event proportions, grouped into deciles of predicted risk; points indicate decile-specific observed proportions and the solid line connects adjacent deciles. The dashed 45° line represents perfect calibration.

Supplementary Material 5: Supplementary Figure 2. Adjusted odds ratios for threshold-defined NIBP measurement gaps (post-alert vs pre-alert). Forest plot showing adjusted odds ratios (ORs) with 95% confidence intervals (horizontal bars) for experiencing at least one noninvasive blood pressure (NIBP) measurement gap of ≥7 min (gap7), ≥10 min (gap10; primary outcome), ≥15 min (gap15), and ≥20 min (gap20) during an anesthetic case (N=34,233). Adjusted ORs (95% CI) were 0.905 (0.760–1.080) for ≥7 min, 0.673 (0.499–0.906) for ≥10 min, 0.613 (0.345–1.088) for ≥15 min, and 0.581 (0.237–1.427) for ≥20 min. ORs at all thresholds were estimated using standard multivariable logistic regression with the same prespecified covariates. The vertical dashed line indicates no between-period difference (OR=1.0); values <1.0 indicate fewer threshold-defined gaps in the post-alert period. Events (cases meeting each threshold) were 5,997 (gap7), 1,890 (gap10), 524 (gap15), and 232 (gap20).

## Data Availability

The datasets generated and/or analyzed during the current study are not publicly available due to institutional policy and restrictions related to patient privacy but are available from the corresponding author on reasonable request, subject to approval by the institutional review board and completion of a data-use agreement.

## Abbreviations

AIMS: Anesthesia information management system
ASA-PS: American Society of Anesthesiologists Physical Status
CI: Confidence interval
IQR: Interquartile range
MAC: Monitored anesthesia care
NIBP: Noninvasive blood pressure
OR: Odds ratio
ORSYS: Operating room information system
PGY: Postgraduate year
SBP: Systolic blood pressure
SD: Standard deviation
SMD: Standardized mean difference
TIVA: Total intravenous anesthesia
AUC: Area under the receiver operating characteristic curve
