# Supplementary material for: Automated alerts and intraoperative noninvasive blood pressure monitoring gaps: a retrospective before-and-after study
Source: BMC Anesthesiol. 2026 Jun 12;26:508. doi: 10.1186/s12871-026-04023-3 (PMC13491824; doi:10.1186/s12871-026-04023-3)
Supplement: Supplementary file 1 [file 12871_2026_4023_MOESM1_ESM.docx]

**Supplementary Table 1.** Distribution of maximum case-level NIBP intervals (minutes) by alert period. Data are summarized as mean (SD), median (IQR), 90th/95th/99th percentiles, and range (minimum–maximum). NIBP, noninvasive blood pressure; SD, standard deviation; IQR, interquartile range; P90/P95/P99, 90th/95th/99th percentiles.

| **Group** | **n** | **Mean ± SD** | **Median [IQR]** | **P90** | **P95** | **P99** | **min** | **max** |
| --- | --- | --- | --- | --- | --- | --- | --- | --- |
| Overall | 34233 | 5.67 ± 2.97 | 5.00 [5.00, 6.00] | 7 | 10 | 17 | 1 | 89 |
| Pre-alert | 15186 | 5.96 ± 3.37 | 5.00 [5.00, 6.00] | 8 | 10 | 19 | 2 | 89 |
| Post-alert | 19047 | 5.44 ± 2.59 | 5.00 [5.00, 6.00] | 7 | 9 | 15 | 1 | 68 |
