# Supplementary material for: Automated alerts and intraoperative noninvasive blood pressure monitoring gaps: a retrospective before-and-after study
Source: BMC Anesthesiol. 2026 Jun 12;26:508. doi: 10.1186/s12871-026-04023-3 (PMC13491824; doi:10.1186/s12871-026-04023-3)
Supplement: Supplementary file 2 [file 12871_2026_4023_MOESM2_ESM.docx]

**Supplementary table** **2.** Gamma GLM for maximum case-level NIBP interval. A multivariable generalized linear model assuming a Gamma distribution with a log link was used to evaluate the association between implementation of the NIBP gap alert and the maximum case-level NIBP measurement interval (gaptime) among all cases. The primary exposure was the alert period (post-alert vs pre-alert). Covariates included age, sex, surgical department, ASA-PS, primary anesthesia provider, type of anesthesia, intraoperative patient position, surgical urgency, duration of anesthesia, arterial line use in the preceding case, and calendar time modeled as elapsed days using a natural cubic spline with 3 degrees of freedom. Results are presented as exponentiated coefficients [exp(β)], interpreted as the ratio of the expected gaptime relative to the reference category, with 95% confidence intervals (CI). NIBP, noninvasive blood pressure; ASA-PS, American Society of Anesthesiologists Physical Status; TIVA, total intravenous anesthesia; MAC, monitored anesthesia care.

| Variable | Reference | exp(β) | exp(β) 95% CI  (lower, upper) | P value |
| --- | --- | --- | --- | --- |
| NIBP alert  Post-alert | Pre-alert | 0.951 | 0.923, 0.980 | 0.001 |
| Age | Per 1-year increase | 1.000 | 1.000, 1.000 | 0.727 |
| Sex Male | Female | 0.992 | 0.981, 1.004 | 0.187 |
| Department of surgery  Orthopedic  Plastic  Obstetrics and Gynecology  Gastrointestinal  Pediatric  Urology  Breast and Endocrine  Hepato-Biliary-Pancreatic  Ophthalmology  Dental and Oral  Neurosurgery  Dermatology  Psychiatry  Others | Otorhinolaryngology | 1.042  1.047  0.986  0.980  1.032  0.981  1.101  0.970  1.032  0.951  1.064  1.042  0.705  1.039 | 1.023, 1.060  1.026, 1.068  0.960, 1.012  0.957, 1.004  1.004, 1.060  0.953, 1.010  1.073, 1.130  0.944, 0.997  0.990, 1.077  0.915, 0.989  1.016, 1.112  0.987, 1.099  0.680, 0.740  0.991, 1.090 | <0.001  <0.001  0.280  0.095  0.023  0.199  <0.001  0.031  0.136  0.012  0.008  0.134  <0.001  0.113 |
| ASA-PS II ≥ III | ASA-PS I | 1.017  1.068 | 1.004, 1.029  1.041, 1.095 | 0.010  <0.001 |
| Primary anesthesia provider Resident + attending Intern + attending | Attending | 1.025  0.999 | 1.011, 1.039  0.986, 1.013 | <0.001  0.951 |
| Type of anesthesia  TIVA Regional MAC | Volatile | 1.018  1.058  0.988 | 1.003, 1.033  1.021, 1.096  0.877, 1.112 | 0.015  0.002  0.839 |
| Position  Lithotomy  Lateral  Prone  Upright | Supine | 0.982  1.122  1.448  1.327 | 0.957, 1.008  1.089, 1.156  1.404, 1.492  1.259, 1.399 | 0.185  <0.001  <0.001  <0.001 |
| Surgical urgency  Emergency | Elective | 0.975 | 0.953, 0.998 | 0.031 |
| Duration of anesthesia | Per 1-min increase | 1.001 | 1.001, 1.001 | <0.001 |
| Arterial line use in the preceding case  Yes | No | 1.058 | 1.044, 1.072 | <0.001 |
