# Supplementary material for: Automated alerts and intraoperative noninvasive blood pressure monitoring gaps: a retrospective before-and-after study
Source: BMC Anesthesiol. 2026 Jun 12;26:508. doi: 10.1186/s12871-026-04023-3 (PMC13491824; doi:10.1186/s12871-026-04023-3)
Supplement: Supplementary file 3 [file 12871_2026_4023_MOESM3_ESM.docx]

**Supplementary table** **3**. Multivariable model for ≥20% SBP change across maximum NIBP gap. A multivariable logistic regression model was used to evaluate factors associated with ≥20% SBP change across maximum NIBP gap, defined as an absolute relative change of systolic blood pressure (SBP) of ≥20% across the maximum intraoperative noninvasive blood pressure (NIBP) measurement gap { |SBP_after − SBP_before| / SBP_before ≥ 0.20 }. The primary exposure was the alert period (post-alert vs pre-alert). Covariates included age, sex, surgical department, ASA-PS, primary anesthesia provider, type of anesthesia, surgical urgency, duration of anesthesia, intraoperative patient position, arterial line use in the preceding case, and calendar time modeled as elapsed days using a natural cubic spline with 3 degrees of freedom. Results are presented as odds ratios (OR) with 95% confidence intervals (CI). SBP, systolic blood pressure; NIBP, noninvasive blood pressure; ASA-PS, American Society of Anesthesiologists Physical Status; TIVA, total intravenous anesthesia; MAC, monitored anesthesia care.

| Variable & Subgroup | Reference | Odds ratio | Odds ratio 95% CI  (lower, upper) | P value |
| --- | --- | --- | --- | --- |
| NIBP alert  Post-alert | Pre-alert | 0.796 | 0.699, 0.907 | <0.001 |
| Age | Per 1-year increase | 1.022 | 1.021, 1.024 | <0.001 |
| Sex  Male | Female | 0.745 | 0.708, 0.784 | <0.001 |
| Department of surgery Orthopedic  Plastic  Obstetrics and Gynecology  Gastrointestinal  Pediatric  Urology  Breast and Endocrine  Hepato-Biliary-Pancreatic  Ophthalmology  Dental and Oral  Neurosurgery  Dermatology  Psychiatry  Others | Otorhinolaryngology | 1.442  0.926  0.940  1.004  0.977  0.962  1.028  1.102  0.842  0.850  1.278  0.967  0.912  0.908 | 1.334, 1.558  0.849, 1.010  0.841, 1.051  0.906, 1.113  0.865, 1.103  0.850, 1.091  0.920, 1.150  0.980, 1.241  0.699, 1.012  0.720, 1.004  1.020, 1.526  0.767, 1.220  0.762, 1.093  0.738, 1.118 | <0.001  0.082  0.276  0.932  0.708  0.550  0.622  0.105  0.067  0.056  0.031  0.777  0.319  0.364 |
| ASA-PS II ≥ III | ASA-PS I | 1.042 0.988 | 0.987, 1.099 0.885, 1.104 | 0.133 0.836 |
| Primary anesthesia provider Resident + attending Intern + attending | Attending | 1.021 0.977 | 0.962, 1.084 0.921, 1.036 | 0.489 0.438 |
| Type of anesthesia  TIVA Regional MAC | Volatile | 1.000  0.200 0.234 | 0.938, 1.065 0.168, 0.239 0.132, 0.413 | 0.995 <0.001 <0.001 |
| Patient position  Lithotomy  Lateral  Prone  Upright | Supine | 1.099  1.008  0.591  0.584 | 0.982, 1.228  0.882, 1.151  0.515, 0.677  0.465, 0.734 | 0.100  0.911  <0.001  <0.001 |
| Surgical urgency  Emergency | Elective | 1.067 | 0.967, 1.177 | 0.193 |
| Duration of anesthesia | Per 1-min increase | 1.000 | 1.000, 1.001 | 0.058 |
| Arterial line use in the preceding case  Yes | No | 1.016 | 0.960, 1.074 | 0.590 |
